# Comparison of Gemcitabine Plus Cisplatin vs. Docetaxel Plus Fluorouracil Plus Cisplatin Palliative Chemotherapy for Metastatic Nasopharyngeal Carcinoma

**DOI:** 10.3389/fonc.2020.01295

**Published:** 2020-08-06

**Authors:** Xue-Song Sun, Xiao-Hao Wang, Sai-Lan Liu, Dong-Hua Luo, Rui Sun, Li-Ting Liu, Shan-Shan Guo, Qiu-Yan Chen, Lin-Quan Tang, Hai-Qiang Mai

**Affiliations:** ^1^Sun Yat-sen University Cancer Center, Guangzhou, China; ^2^State Key Laboratory of Oncology in South China, Guangzhou, China; ^3^Collaborative Innovation Center for Cancer Medicine, Guangdong Key Laboratory of Nasopharyngeal Carcinoma Diagnosis and Therapy, Guangzhou, China; ^4^Department of Nasopharyngeal Carcinoma, Sun Yat-sen University Cancer Center, Guangzhou, China

**Keywords:** nasopharyngeal carcinoma, palliative chemotherapy, GP regimen, TPF regimen, survival

## Abstract

**Objective:** Our study aimed to compare the efficacy and toxicity of two chemotherapy regimens, gemcitabine plus cisplatin (GP) vs. docetaxel plus, fluorouracil plus cisplatin (TPF), in metastatic nasopharyngeal carcinoma (NPC) patients.

**Methods:** We retrospectively enrolled metastatic NPC patients between July 2006 and December 2016 who were treated with TPF or GP palliative chemotherapy (PCT). The association between the PCT regimens and survival conditions was evaluated by log-rank tests and the Cox proportional hazards model. A cohort was created using propensity score matching with the ratio of 1:1 to clarify the results of the multivariable Cox regression analyses. Overall survival (OS) was the primary endpoint.

**Results:** Of 266 eligible patients, 186 and 80 patients, respectively, received TPF and GP regimen. No significant difference was demonstrated in the survival rate between the GP and TPF groups (3-year OS: 52.6 vs. 50.3%; *P* = 0.929). However, multivariable analysis suggested receiving GP as an independent protective factor (hazard ratio, 0.864; 95% confidence interval, 0.753–0.992; *P* = 0.042). In the matched cohort, treatment with GP was also associated with a significantly higher OS (3-year OS: 52.6 vs. 35.6%, *P* = 0.042). Subgroup analysis indicated that the superiority of GP reflected in patients with secondary metastases rather than primary metastases. The incidence of grade 3 to 4 treatment-related toxicity was more common in the TPF group than in the GP group.

**Conclusion:** Our study suggested that GP might be superior to TPF for metastatic NPC patients, especially those with secondary distant metastases. Further studies are necessary to validate our results.

## Introduction

Nasopharyngeal carcinoma (NPC) is a kind of malignancy arising from the nasopharyngeal mucosal lining. Different from other head and neck cancers, the incidence of NPC is obviously unbalanced across the world, with the highest incidence rate observed in South China (1). Because of its radiosensitivity, radiotherapy (RT) with or without chemotherapy is the standard treatment method for non-metastatic NPC (2, 3). Nowadays, distant metastases have become the main treatment failure and cause of death in NPC (4). Besides, approximately 15% of patients are detected to have distant metastases at the point of primary diagnosis (5). Once distant lesions are present, the prognosis is poor, and treatment mainly relies on systemic palliative chemotherapy (PCT) (6).

Various of platinum-based PCTs are widely applied in metastatic patients (7–9). However, it remains unknown which PCT regimen is the best, considering the trade-off between efficacy and toxicity. A randomized trial has verified that patients receiving gemcitabine plus cisplatin (GP) achieved better survival outcomes when compared with fluorouracil plus cisplatin (PF) (10). Meanwhile, in head and neck cancer, several large-scale phase III trials have shown the statistically significant survival benefits of adding docetaxel to the PF (TPF) induction regimen, and the superiority has also been observed in locally advanced NPC (11–15). Therefore, the comparison between the GP and TPF regimens is of great clinical significance. For non-metastatic NPC, Zhu et al. (16) demonstrated that the GP regimen was equivalent to TPF in treatment outcomes but with less toxicity. Up to now, no study has compared the efficacy and toxicity between TPF and GP in metastatic NPC.

In this study, we retrospectively enrolled 266 metastatic NPC patients receiving TPF or GP regimens. Based on the relatively large sample size, we compared the survival condition and acute toxicity of patients between these two PCT groups, in order to provide important information for determining the proper PCT regimen for metastatic NPC patients.

## Materials and Methods

### Patient Selection and Pretreatment Evaluation

**Figure 1:**
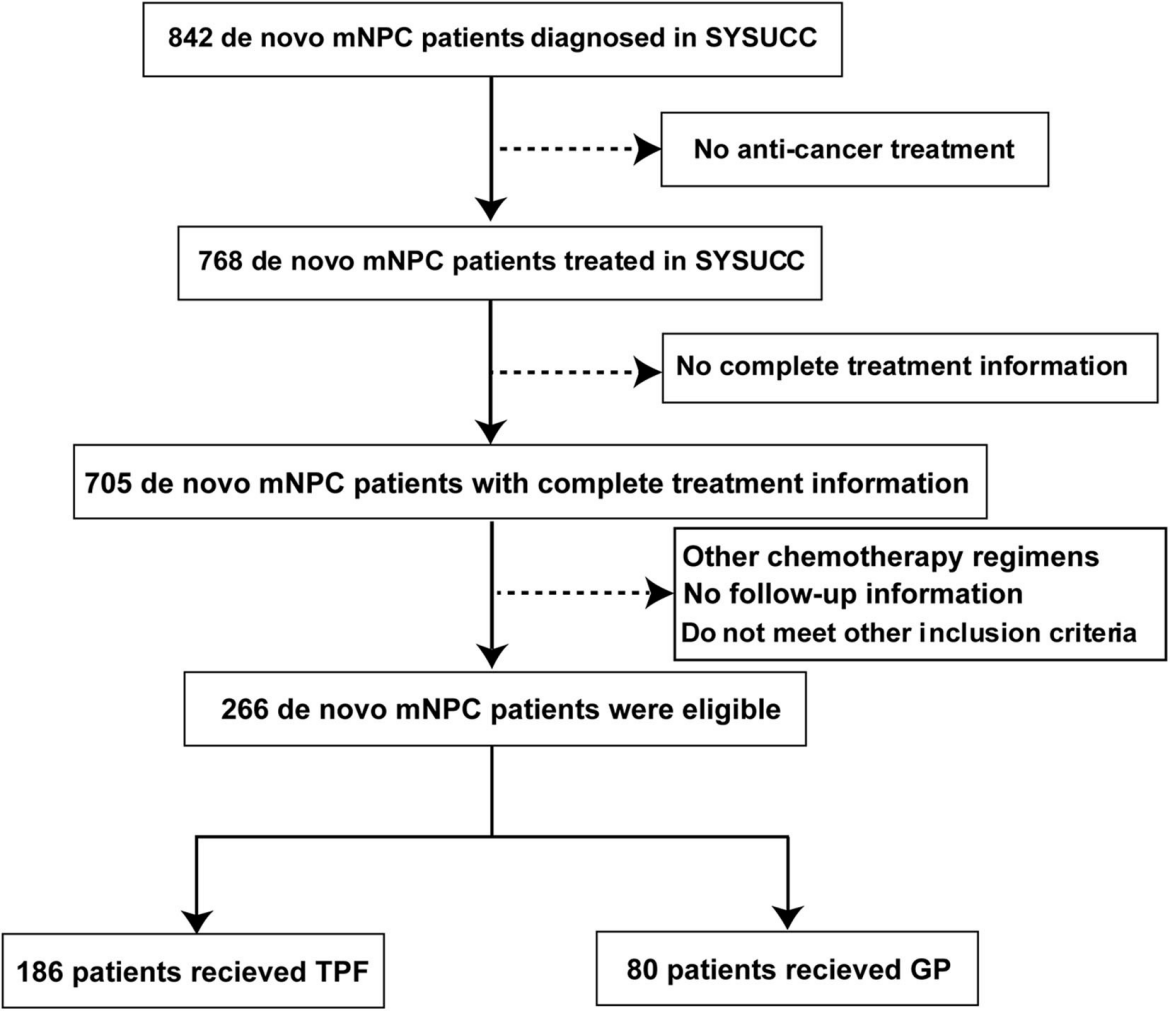
Flowchart of study patient inclusion. mNPC, metastatic nasopharyngeal carcinoma; GP, gemcitabine plus cisplatin; TPF, docetaxel plus fluorouracil plus cisplatin.

From July 2006 to December 2016, a total of 266 metastatic NPC patients treated in the Sun Yat Sen University Cancer Center were retrospectively enrolled into this study. The eligibility criteria were as follows: (1) biopsy-proven NPC; (2) evidence of distant metastasis confirmed by pathology or imaging examinations; (3) received GP or TPF regimen as the first-line treatment; (4) complete accessible treatment records; (5) aged ≥18 years; (6) adequate organ functions; (7) Karnofsky performance score >70; and (8) no pregnancy, lactation, or second malignancy. Our study was approved by the Research Ethics Committee of our center. The flowchart is described in Figure 1.

Pretreatment evaluations were performed in every enrolled patient, including physical examinations, fiberoptic nasopharyngoscopy, magnetic resonance imaging/computed tomography (CT) of the head and neck, and whole-body examination including chest X-rays/chest CT, abdominal sonography/abdominal CT, and bone scans. The positron emission tomography–CT was selectively performed based on clinician judgment. Complete blood count and biochemical profiles were also required.

### Chemotherapy and Local Treatment

The GP and TPF regimens were administered as the first-line treatment in this study. The detailed regimens were as follows: TPF: docetaxel [60 mg/m^2^ docetaxel intravenously [IV] given on day 1], cisplatin (20–25 mg/m^2^ IV on days 1–3), and 5-fluorouracil (500–800 mg/m^2^ continuous IV infusion for 24 h on days 1–5); GP: gemcitabine (800–1,000 mg/m^2^ IV on days 1 and 8) and cisplatin (20–30 mg/m^2^ IV on days 1–3). The cumulative dose of cisplatin was 60 to 75 mg/m^2^ and 70 to 85 mg/ m^2^ in TPF and GP regimens, respectively. Based on the treatment principle in our center, patients with metastatic NPC were given four to six cycles of PCT. The treatment would be terminated or changed under the following conditions: disease progression, death, occurrence of intolerable toxicities, or at patient's request. For patients with limited or localized metastatic lesions, local treatment, such as surgery, RT, or interventional ablative therapy, was considered according to the clinician's judgment.

### Outcome and Follow-Up

Overall survival (OS) was the primary endpoint, calculated from the date of diagnosis to death. Patients who were lost to follow-up or alive had their follow-ups censored at the last visit. Patients were routinely followed up every 3 months during the first year and every 6 months thereafter until death. Magnetic resonance imaging or CT of the head and neck, chest X-rays/CT, abdominal sonography/CT, and bone scans were generally performed.

## Statistical Analyses

The patients' baseline characteristics between the two groups were evaluated by the χ^2^-test. Kaplan–Meier survival curves were used to estimate the OS curves, and the difference was compared by log-rank test. A multivariable Cox regression model was utilized to calculate the hazard ratios (HRs) and 95% confidence intervals (CIs). The propensity score matching (PSM) method was applied to balance potential confounders, using a 1:1 matching protocol with a greedy-matching algorithm, and the caliper width equaled 0.05. The following factors were included in the matching process: age, gender, smoking history, primary or secondary metastases, number of metastatic organs, chemotherapy cycles, and local treatment. To evaluate the predictive accuracy of Epstein-Barr virus (EBV) DNA, the time-dependent (3-year) receiver operating characteristic (ROC) analysis was applied. The area under the ROC curve (AUC) was calculated to assess the sensitivity and specificity of EBV DNA to predict death. The cutoff value of EBV DNA was selected based on the minimum P (highest χ^2^) value defined by log-rank test (17). Kaplan–Meier survival curve and Cox regression model were used to explore the prognostic value of EBV DNA. Interaction analysis based on the Cox proportional hazards model was performed between chemotherapy regimens and the state of metastasis, primary or secondary. The efficacy of two regimens was compared in the subgroups of either primary or secondary disease. All analyses were two-sided, and a two-tailed *P* < 0.05 indicated a difference with statistical significance. The statistical analyses were performed using SPSS for Mac version 23.0 (SPSS Inc., Chicago, IL, USA), R 3.5.1 (R Project, Vienna, Austria) and X-tile software (V.3.6.1; Yale University, New Haven, CT, USA).

## Results

### Patient Characteristics

Of the entire cohort, the median age was 47 years with a male-to-female ratio of 4.5:1. There were 186 patients and 80 patients, respectively, assigned to the TPF and GP groups. As shown in Table 1, there was a significantly higher proportion of patients with multiple metastatic organs (40.0 vs. 25.8%, *P* = 0.028) and secondary metastasis in the GP group (62.5 vs. 27.4%, *P* < 0.001). The median accumulative cisplatin dose was 320 mg/m^2^. Patients in the GP group received higher intensity of cisplatin treatment (*P* < 0.001). Other baseline characteristics were in good balance between the two treatment groups.

**Table 1:** Clinical characteristics in whole cohort.

**Characteristics**	**TPF (*n* = 186)**	**GP (*n* = 80)**	***P***
	**No. (%)**	**No. (%)**	
**Age (years)**			
≤ 47	93 (50.0%)	42 (52.5%)	0.789
>47	93 (50.0%)	38 (47.5%)	
**Gender**			
Male	154 (82.8%)	64 (80.0%)	0.604
Female	32 (17.2%)	16 (20.0%)	
**Smoking history**			
Non-smokers	99 (53.2%)	48 (60.0%)	0.348
Smokers	87 (46.8%)	32 (40.0%)	
**Time order**			
Primary metastases	135 (72.6%)	30 (37.5%)	<0.001
Secondary metastases	51 (27.4%)	50 (62.5%)	
**No. of metastatic organs**			
Oligo	138 (74.2%)	48 (60.0%)	0.028
Multiple	48 (25.8%)	32 (40.0%)	
**Chemotherapy cycles**			
≤ 4	85 (45.7%)	33 (41.3%)	0.591
>4	101 (54.3%)	47 (58.8%)	
**Total platinum dose (mg/m**^**2**^**)**			
Median (range)	300 (60–600)	420 (80–800)	<0.001*
**Local treatment of metastases**			
No	157 (84.4%)	73 (91.3%)	0.172
Yes	29 (15.6%)	7 (8.8%)	
**EBV DNA^#^**			
≤ 30,000 copies/mL	69 (43.9%)	19 (42.2%)	0.866
>30,000 copies/mL	88 (56.1%)	26 (57.8%)	

*TPF, cisplatin plus docetaxel plus 5-fluorouracil; GP, cisplatin plus gemcitabine; EBV, Epstein–Barr virus*.

The P-value was calculated using the Pearson χ^2^-test and t-test (*).

# *Two hundred two patients had the data of EBV DNA*.

### Survival Analysis

**Figure 2:**
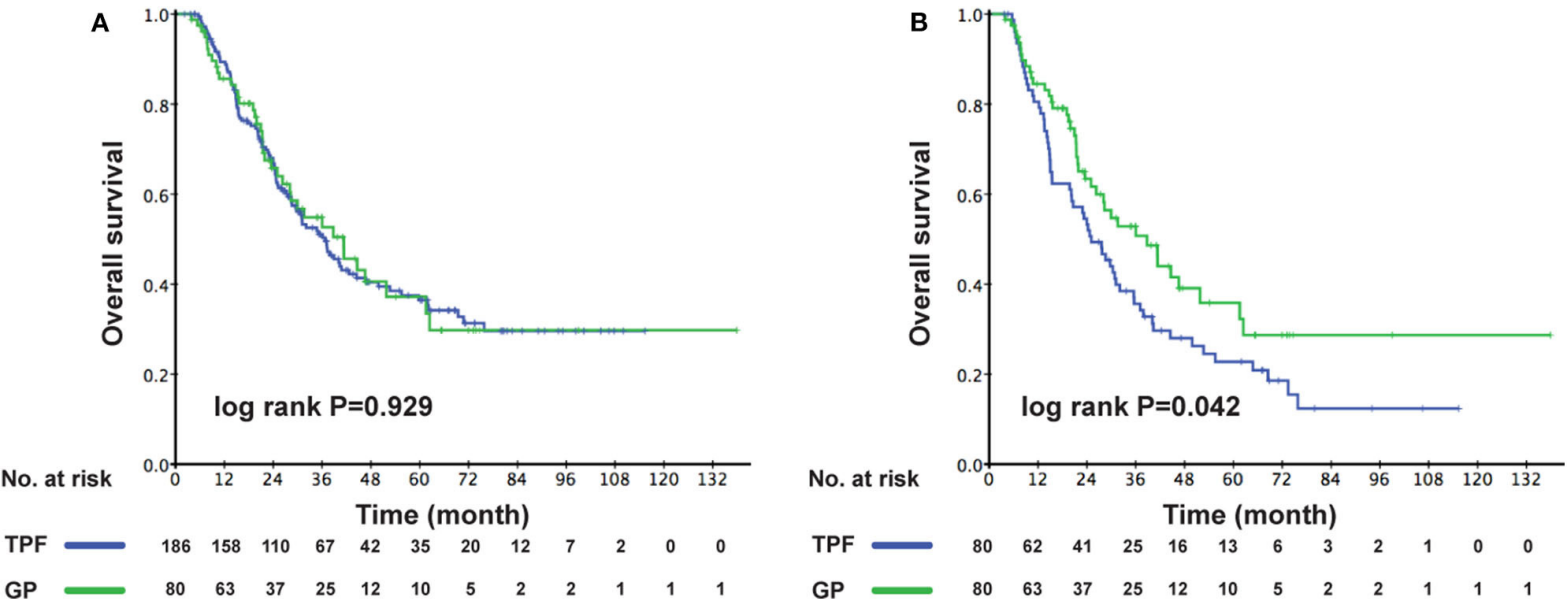
Kaplan–Meier OS curves for the metastatic patients receiving GP and TPF **(A)** in the whole cohort; **(B)** in the PSM cohort. OS, overall survival; GP, gemcitabine plus cisplatin; TPF, docetaxel plus fluorouracil plus cisplatin; PSM, propensity score matching.

The cutoff of data for OS analysis was on July 1, 2019. With a median follow-up of 26.7 months (range, 1.2–137.9 months), 143 patients (53.8%) died during follow-up, including 104/186 (55.9%) in the TPF group and 39/80 (48.6%) in the GP group. The 1-, 3-, and 5-year survival rates for the entire cohort were 87.9, 51.6, and 36.6%, respectively. The OS rates were similar between patients who received GP and TPF (3-year OS: 52.6 vs. 50.3%; *P* = 0.929) (Figure 2A). However, after adjusting for other variables, GP was shown to be an independent protective factor (GP vs. TPF: HR, 0.864; 95% CI, 0.753–0.992; *P* = 0.042) in multivariable analysis (Table 2). Besides, patients with secondary metastases (HR, 1.567; 95% CI, 1.064–2.308; *P* = 0.023) and multiple metastatic organs (HR, 2.137; 95% CI, 1.459–3.129; *P* < 0.001) were also associated with worse survival outcomes. To further clarify the results of the multivariable analyses, a cohort of 160 patients were created using the PSM. All of the patients in GP group find a close unique match in TPF group. As shown in Table S1, the characteristics between the two treatment groups were well-balanced. We then compared patients' survival in the matched cohort and found that the application of GP regimen contributed to survival prolongation, with a significantly higher OS rate (3-year OS: 52.6 vs. 35.6%, *P* = 0.042) (Figure 2B).

**Table 2:** Multivariable analysis.

**Characteristic**	**Model 1**			**Model 2^#^**			**Model 3**		
	**HR**	**95% CI**	***P***	**HR**	**95% CI**	***P***	**HR**	**95% CI**	***P***
Age (years)	1.320	0.931–1.870	0.119	1.390	0.897–2.155	0.140	1.307	0.927–1.844	0.126
Gender	1.197	0.791–1.810	0.394	0.942	0.533–1.663	0.836	1.139	0.754–1.721	0.537
Smoking history	1.052	0.749–1.477	0.769	1.282	0.837–1.963	0.254	1.127	0.798–1.591	0.498
Time order	1.567	1.064–2.308	0.023	1.603	1.109–2.548	0.027	2.801	1.557–5.040	0.001
No. of metastatic organs	2.137	1.459–3.129	<0.001	2.997	1.856–4.838	<0.001	2.259	1.540–3.315	<0.001
Chemotherapy cycles	1.063	0.665–1.700	0.798	1.050	0.671–1.642	0.832	1.052	0.734–1.507	0.783
Local treatment of metastases	0.656	0.379–1.138	0.134	0.902	0.477–1.709	0.753	0.628	0.364–1.082	0.094
Total platinum dose	1.007	0.599–1.696	0.978	0.666	0.340–1.305	0.237	1.024	0.610–1.719	0.928
Chemotherapy regimens	0.864	0.753–0.992	0.042	0.752	0.700–0.978	0.022	0.932	0.747–1.157	0.552
EBV DNA level				2.159	1.381–3.373	0.001			
Chemotherapy regimens * time order							0.740	0.568–0.966	0.026

*HR, hazard ratio; CI, confidence interval; TPF, cisplatin plus docetaxel plus 5-fluorouracil; GP, cisplatin plus gemcitabine, EBV, Epstein–Barr virus*.

*A Cox proportional hazards model was used to perform multivariable analyses. All variables were transformed into categorical variables. HRs were calculated for age (years) (>47 vs. ≤ 47); gender (male vs. female); smoking history (smokers vs. non-smokers); time order (secondary metastases vs. primary metastases); number of metastatic organs (multiple vs. oligo); chemotherapy cycles (>4 vs. ≤ 4); local treatment of metastases (yes vs. no); total platinum dose (≥320 vs. <320 mg/m^2^); and chemotherapy regimens (GP vs. TPF); EBV DNA (>30,000 copies/mL vs. ≤ 30,000 copies/mL)*.

# *Two hundred two patients who had the data of EBV DNA were involved in Model 2*.

**Figure 3:**
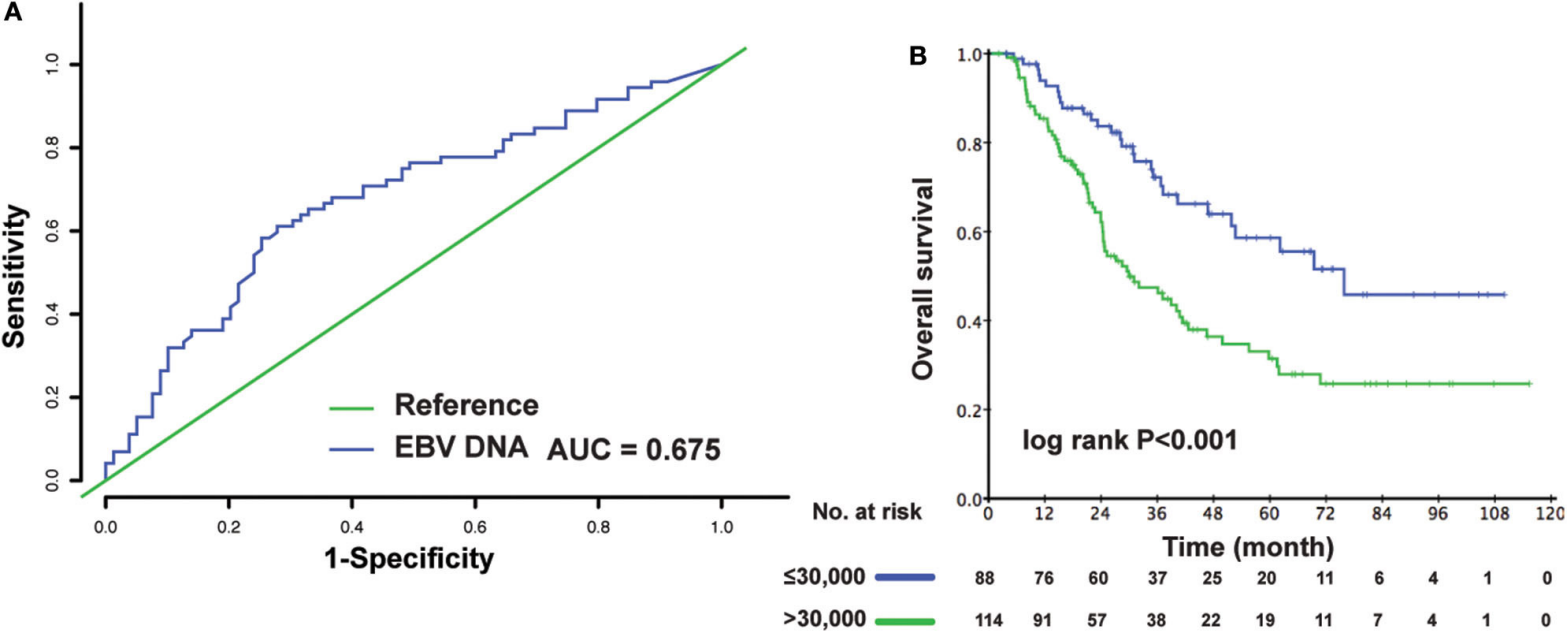
The time-dependent 3-year ROC curve **(A)**. Kaplan–Meier OS curves for the metastatic patients in different EBV DNA level **(B)**. One hundred fifty-two patients with follow-up information of more than 3 years were involved in the ROC analysis.

Two hundred two patients had EBV DNA levels measured at admission. We analyzed the effect of EBV DNA on prognosis among these patients. EBV DNA had a satisfactory value in the prediction of death concerning time-dependent 3-year ROC (AUC = 0.675) (Figure 3A). The optimal cutoff value of EBV DNA was 30,300 copies/mL, which was generated by X-tile plots. For better clinical application, the cutoff value was rounded to 30,000 copies/mL. As shown in Figure 3B, lower EBV DNA level (≤ 30,000 copies/mL) was significantly associated with better survival condition (3-year OS: 70.2 vs. 46.2%, *P* < 0.001). An additional multivariable model was made in these patients, and the EBV DNA level was found to be an independent risk factor for OS (>30,000 vs. ≤30,000 copies/mL: HR, 2.159; 95% CI, 1.381–3.373; *P* = 0.001) (Table 2).

### Subgroup Analysis

**Figure 4:**
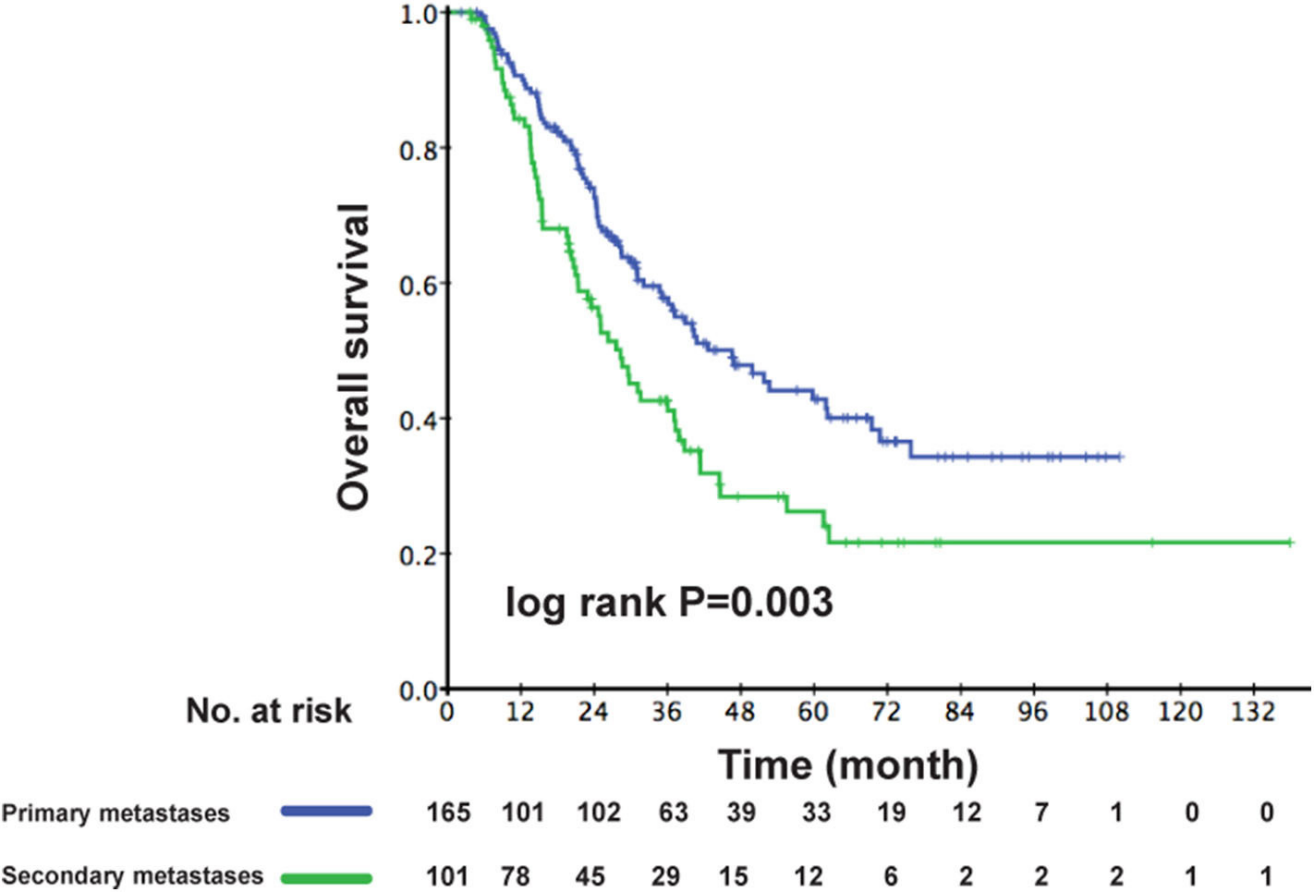
Kaplan–Meier OS curves for patients with primary metastases and secondary metastases. OS, overall survival.

**Figure 5:**
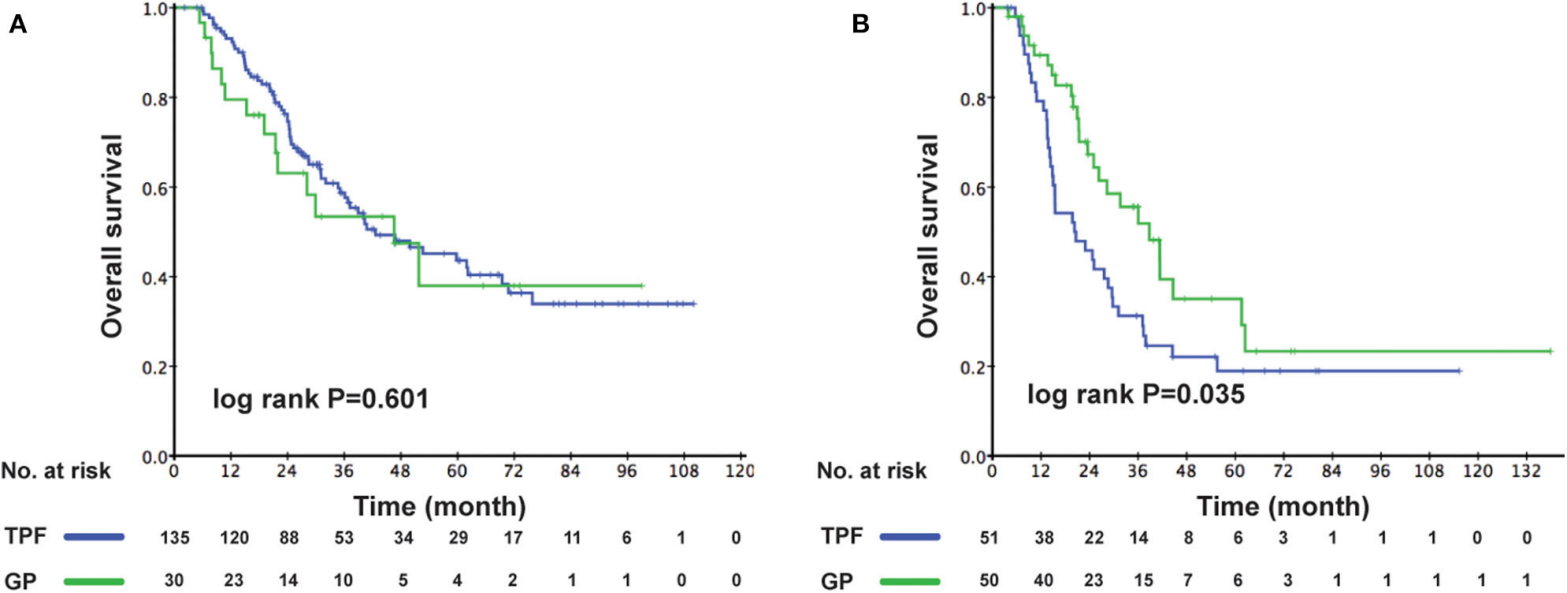
Comparison of OS for patients in the GP and TPF groups: **(A)** patients with primary metastases and **(B)** patients with secondary metastases. OS, overall survival; GP, gemcitabine plus cisplatin; TPF, docetaxel plus fluorouracil plus cisplatin.

As the multivariable analysis demonstrated, whether metastases occurred primarily or secondarily was an independent prognostic factor. The 3-year OS rates were 56.9 and 41.1%, respectively, for primary and secondary disease (*P* = 0.003); the Kaplan–Meier curves are shown in Figure 4. We further preformed an interaction analysis in another Cox model (Model 3). As shown in Table 2, an interaction effect existed between chemotherapy regimens and the state of metastases (*P* = 0.026). Therefore, a subgroup analysis was conducted to explore the impact of different PCT regimens based on the state of metastasis. The clinical characteristics of patients with primary/secondary metastases were shown in Table 3. Interestingly, the superiority of GP in OS was observed only in patients with secondary metastases (3-year OS: 51.9 vs. 29.0%; *P* = 0.035), whereas no significant difference was found in primary metastatic NPC patients (3-year OS: 53.4 vs. 57.6%; *P* = 0.601) (Figure 5). Multivariable analysis also indicated that compared with TPF, the administration of GP was a protective factor for secondary metastatic NPC (HR, 0.797; 95% CI, 0.614–0.982; *P* = 0.022), but not for primary metastatic NPC patients (HR, 1.069; 95% CI, 0.855–1.337; *P* = 0.557) (Table 4).

**Table 3:** Clinical characteristics of patients in primary/secondary metastases subgroups.

	**Primary metastases** ***n*** **= 165**			**Secondary metastases** ***n*** **= 101**		
**Characteristic**	**TPF (*n* = 135)**	**GP (*n* = 30)**	***P***	**TPF (*n* = 51)**	**GP (*n* = 50)**	***P***
**Age (years)**						
≤ 47	63 (46.7%)	12 (40.0%)	0.549	30 (58.8%)	30 (60.0%)	1.000
>47	72 (53.3%)	18 (60.0%)		21 (41.2%)	20 (40.0%)	
**Gender**						
Male	113 (83.7%)	24 (80.0%)	0.788	28 (54.9%)	27 (54.0%)	1.000
Female	22 (16.3%)	6 (20.0%)		23 (45.1%)	23 (46.0%)	
**Smoking history**						
Non-smokers	71 (52.6%)	21 (70.0%)	0.104	28 (54.9%)	27 (54.0%)	1.000
Smokers	64 (47.4%)	9 (30.0%)		23 (45.1%)	23 (46.0%)	
**No. of metastatic organs**						
Oligo	106 (78.5%)	22 (73.3%)	0.629	32 (62.7%)	26 (52.0%)	0.318
Multiple	29 (21.5%)	8 (26.7%)		19 (37.3%)	24 (48.0%)	
**Chemotherapy cycles**						
≤ 4	113 (83.7%)	29 (96.7%)	0.080	44 (86.3%)	44 (88.0%)	1.000
>4	22 (16.3%)	1 (3.3%)		7 (13.7%)	6 (12.0%)	
**Local treatment of metastases**						
No	61 (45.2%)	13 (43.3%)	1.000	24 (47.1%)	20 (40.0%)	0.549
Yes	74 (54.8%)	17 (56.7%)		27 (52.9%)	30 (60.0%)	

*TPF, cisplatin plus docetaxel plus 5-fluorouracil; GP, cisplatin plus gemcitabine*.

*The P-value was calculated using the Pearson χ^2^-test*.

**Table 4:** Multivariable analyses in primary metastatic and secondary metastatic patients.

	**Primary metastases**			**Secondary metastases**		
**Characteristic**	**HR**	**95% CI**	***P***	**HR**	**95% CI**	***P***
Age (years)	1.259	0.788–2.012	0.335	1.398	0.832–2.349	0.206
Gender	0.987	0.539–1.809	0.967	1.375	0.760–2.486	0.292
Smoking history	1.298	0.813–2.071	0.275	0.923	0.546–1.561	0.764
No. of metastatic organs	2.780	1.638–4.718	<0.001	1.845	1.051–3.239	0.033
Chemotherapy cycles	1.265	0.644–2.484	0.495	0.768	0.396–1.491	0.435
Local treatment of metastases	0.838	0.406–1.730	0.632	0.432	0.183–1.017	0.055
Total platinum dose	0.993	0.485–2.034	0.984	0.984	0.457–2.117	0.967
Chemotherapy regimens	1.069	0.855–1.337	0.557	0.797	0.614–0.982	0.022

*HR, hazard ratio; CI, confidence interval; TPF, cisplatin plus docetaxel plus 5-fluorouracil; GP, cisplatin plus gemcitabine*.

*Data were obtained from all 266 patients included in the study*.

*A Cox proportional hazards model was used to perform multivariable analyses. All variables were transformed into categorical variables. HRs were calculated for age (years) (>47 vs. ≤47); gender (male vs. female); smoking history (smokers vs. non-smokers); number of metastatic organs (multiple vs. oligo); chemotherapy cycles (>4 vs. ≤4); local treatment of metastases (yes vs. no); total platinum dose (≥320 vs. <320 mg/m^2^); and chemotherapy regimens (GP vs. TPF)*.

### Toxicity

The differences of grade 3 to 4 adverse events (AEs) between 2 PCT regimens were analyzed in our study (Table 5). The two most common grade 3 to 4 AEs were leukopenia and neutropenia. Besides, a higher frequency of G3 to G4 neutropenia was observed in the TPF group (41.9 vs. 25.0%, *P* = 0.012). Patients receiving GP were inclined to suffer from G3–G4 thrombocytopenia, but with non-statistical significance (15.0 vs. 8.1%, *P* = 0.119). Serious hepatotoxicity and nephrotoxicity were both low in two groups, and no significant differences was found. More G3–G4 mucositis occurred in the TPF group (11.8 vs. 0.0%, *P* < 0.001). No drug-related fatal AEs were reported in the current study.

**Table 5:** Acute toxicities during chemotherapy between the two groups.

	**TPF (*n* = 186)**	**GP (*n* = 80)**	***P***
	**No. (%)**	**No. (%)**	
**Leukocytopenia**			
G0–2	122 (65.6%)	57 (71.3%)	0.395
G3–4	64 (34.4%)	23 (28.8%)	
**Neutropenia**			
G0–2	108 (58.1%)	60 (75.0%)	0.012
G3–4	78 (41.9%)	20 (25.0%)	
**Anemia**			
G0–2	168 (90.3%)	76 (95.0%)	0.235
G3–4	18 (9.7%)	4 (5.0%)	
**Thrombocytopenia**			
G0–2	171 (91.9%)	68 (85.0%)	0.119
G3–4	15 (8.1%)	12 (15.0%)	
**Hepatotoxicity**			
G0–2	179 (96.2%)	78 (97.5%)	0.728*
G3–4	7 (3.8%)	2 (2.5%)	
**Nephrotoxicity**			
G0–2	185 (99.5%)	80 (100.0%)	1.000*
G3–4	1 (0.5%)	0 (0.0%)	
**Mucositis**			
G0–2	164 (88.2%)	80 (10.0%)	<0.001
G3–4	22 (11.8%)	0 (0.0%)	

The P-value was calculated using the Pearson χ^2^-test or Fisher exact test (*).

## Discussion

To our knowledge, this is the first study to compare the efficacy and safety of TPF to GP in metastatic NPC. Compared with TPF, the administration of GP was a protective factor for OS with fewer G3–G4 AEs. According to the results, GP could serve as first-line treatment for metastatic NPC patients, in particular for those with secondary metastases. Prospective and large-sample studies are needed to validate our results.

Nowadays, with the development of RT technology, NPC patients have obtained satisfactory local regional control. Distant metastasis remains a major challenge in the management of NPC and also the leading cause of death (4, 18). Because of the huge tumor burden for metastatic patients, platinum-based systemic palliative therapy has become the main treatment method with objective response rates of 55 to 80% (7–9). However, the duration of response is short, and the long-term survival is still poor (19). Which PCT regimen is the best choice is still under discussion.

For locally advanced NPC, patients receiving TPF achieved better survival when compared with PF and (docetaxel plus cisplatin) TP, which was considered as the most effective chemotherapy regimen (14, 15). Unfortunately, the triple regimen also brought a higher incidence of treatment-related AEs (15). Because multiple cycles of chemotherapy were indispensable, the tolerability of chemotherapy regimens became a big issue faced by metastatic patients. Therefore, an effective and tolerable PCT regimen for these patients was in urgent need.

Gemcitabine is a ribonucleotide reductase inhibitor, which shows a broad-spectrum antitumor activity, including NPC (20). More importantly, gemcitabine can enhance the activity of cisplatin by increasing the formation of DNA adducts induced by platinum and inhibiting the activity of ERCC1, which is an important mechanism of platinum resistance (21). The synergistic effect of cisplatin and gemcitabine has been verified in a variety of human tumor cells *in vitro* (22, 23). At present, this combination is used in the treatment for a variety of malignant tumors (24, 25). Among locally advanced NPC patients, a multicenter phase III trial demonstrated that GP induction chemotherapy (IC) significantly improved the survival condition when compared with concurrent chemoradiotherapy alone (26). Compared with a PF regimen, cost-effectiveness analysis proved that GP was more cost-effective than the traditional regimen (27). Moreover, in patients with recurrent or metastatic NPC, Zhang et al. (10) verified the superiority of GP over PF in terms of efficacy and toxicity. However, no study directly compared the efficacy of GP to TPF, and the latter one has been considered a stronger PCT regimen when compared to PF in metastatic NPC patients.

In the present study, we compared these two PCT regimens in 266 metastatic patients and found that the OS was similar between the two groups (50.3 vs. 52.6%; *P* = 0.929). However, it should be noted that the patient's characteristics were unbalanced between the two groups, and we observed higher proportions of multiple metastatic organs (*P* = 0.028) and secondary metastases in the GP group (*P* < 0.001). After adjusting for important variables, GP was an independent protective factor with a 14.0% lower risk of death as compared to the TPF group in multivariable analysis. In the matched cohort, a higher 3-year OS in the GP group was also achieved (52.6 vs. 35.6%, *P* = 0.042). Comparison between the two regimens indicated that the triple regimen resulted in a higher grade 3–4 AEs in neutropenia (*P* = 0.012) and mucositis (*P* < 0.001). The incidences of grade 3–4 AEs in the GP group were similar to a previous clinical trial (10).

Additionally, our group verified that secondary metastases were associated with worse prognosis than primary metastases. Interaction analysis showed that interaction effect existed between chemotherapy regimens and the state of metastases occurrence. Therefore, we performed subgroup analysis in patients with different state of metastases. Interestingly, the superiority of GP over TPF was consistently seen only in patients with secondary metastases. This result could be partially explained by the following. On the one hand, the oral health status of NPC patients after RT is generally poor, and it can be further aggravated by receiving fluorouracil and cisplatin regimen because it may lead to severe mucositis. As the adverse reactions are intolerable, the patients are more likely to discontinue the treatment (10). In addition, deep vein catheterization for fluorouracil infusion also increases the risk of catheter-related infection and thromboembolism, which also compromised their survival rates (28). These conditions affect the efficacy of TPF regimen. However, as for GP regimen, the most common blood toxicity can be more easily identified and dealt with. On the other hand, fluorouracil or taxol-containing regimens are widely used as the IC for locally advanced NPC. For patients who developed metastases after primary treatment, the previous use of fluorouracil or taxol-containing IC might have led to chemotherapy resistance of the corresponding drugs and will weaken the efficacy of the subsequent palliative TPF regimen to some degrees (29, 30). Unfortunately, as some patients did not receive the initial treatment in our center, the previous treatment details were inaccessible; thus, we could not provide the relevant information in current study.

Our study showed the advantage of GP in improving efficacy and reducing toxicities when compared with TPF in metastatic NPC, and we recommended the use of GP as first-line treatment, especially for metastases after primary treatment. Besides, the administration of GP regimen is also simpler than TPF, and outpatient treatment is feasible, which could reduce costs for patients and the stress of hospital stay.

There were several limitations to the current study. First, this was a retrospective study, and the selective bias was unavoidable. Therefore, we performed multivariable analysis to eliminate confounding factors to some extent, and the well-balanced cohort using the PSM was considered to eliminate potential confounders between patients who received TPF and GP. Besides, it is difficult to determine the accurate disease progression time because of the retrospective design. Therefore, OS was the only endpoint in current study. Second, in view of the different types of failure pattern, cause of death, and prognosis between recurrent and metastatic NPC, patients with local recurrence were not included in this study. Third, some patients only used chest X-ray and abdominal ultrasound to evaluate their metastatic conditions, which offered limited evaluative value to treatment response. Finally, all patients involved in this study were from an endemic area, and the predominant histology is the undifferentiated non-keratinizing carcinoma. Whether the results could be applied to non-endemic areas needs to be verified. A well-designed prospective clinical trial is necessary to validate our results.

## Conclusion

Our study suggests that the GP PCT regimen achieved better efficacy compared to the TPF PCT regimen among patients with secondary metastatic NPC. The incidence of grade 3–4 AEs was relatively lower in the GP group than in the TPF group. Prospective studies are awaited to validate our results.

## Data Availability Statement

The datasets generated for this study are available on request to the corresponding author.

## Ethics Statement

This retrospective study was approved by the Clinical Research Committee of Sun Yat Sen University Cancer Center. The patients/participants provided their written informed consent to participate in this study. Written informed consent was obtained from the individual(s) for the publication of any potentially identifiable images or data included in this article.

## Author Contributions

H-QM, L-QT, and Q-YC: study concepts. X-SS, X-HW, and S-LL: study design. X-SS, X-HW, S-LL, D-HL, RS, L-TL, and S-SG: data acquisition. X-SS, X-HW, and S-LL: quality control of data and algorithms. X-SS, X-HW, RS, D-HL, L-TL, S-SG, and S-LL: data analysis and interpretation. X-SS, X-HW, and S-LL: statistical analysis. X-SS, X-HW, S-LL, RS, D-HL, L-TL, S-SG, H-QM, L-QT, and Q-YC: article preparation. X-SS, X-HW, and S-LL: article editing. X-SS, X-HW, S-LL, RS, D-HL, L-TL, S-SG, L-QT, Q-YC, and H-QM: article review. All authors contributed to the article and approved the submitted version.

## Conflict of Interest

The authors declare that the research was conducted in the absence of any commercial or financial relationships that could be construed as a potential conflict of interest.
