# Supplementary material for: Comparison of Gemcitabine Plus Cisplatin vs. Docetaxel Plus Fluorouracil Plus Cisplatin Palliative Chemotherapy for Metastatic Nasopharyngeal Carcinoma
Source: Front Oncol. 2020 Aug 6;10:1295. doi: 10.3389/fonc.2020.01295 (PMC7425654; doi:10.3389/fonc.2020.01295)
Supplement: Supplementary file 1 [file Table_1.docx]

Table S1. Clinical characteristics in PSM cohort

| **Characteristics** | TPF (n=80) | GP (n=80) | *P*-value |
| --- | --- | --- | --- |
|  | No. (%) | No. (%) |  |
| **Age (years)** |  |  |  |
| ≤47 | 43(53.8%) | 42(52.5%) | 1.000 |
| >47 | 37(46.3%) | 38(47.5%) |  |
| **Gender** |  |  |  |
| Male | 67(83.8%) | 64(80.0%) | 0.682 |
| Female | 13(16.3%) | 16(20.0%) |  |
| **Smoking history** | |  |  |
| Non-smokers | 40(50.0%) | 48(60.0%) | 0.266 |
| Smokers | 40(50.0%) | 32(40.0%) |  |
| **Time order** |  |  |  |
| Primary metastases | 37(46.3%) | 30(37.5%) | 0.336 |
| Secondary metastases | 43(53.8%) | 50(62.5%) |  |
| **Number of metastatic organs** | |  |  |
| Oligo | 53(66.3%) | 48(60.0%) | 0.512 |
| Multiple | 27(33.8%) | 32(40.0%) |  |
| **Chemotherapy cycles** | |  |  |
| ≤4 | 33(41.3%) | 33(41.3%) | 1.000 |
| >4 | 47(58.8%) | 47(58.8%) |  |
| **Local treatment of metastases** | |  |  |
| No | 74(92.5%) | 73(91.3%) | 1.000 |
| Yes | 6(7.5%) | 7(8.8%) |  |

Abbreviations: PSM, propensity score matching; TPF, cisplatin plus docetaxel plus 5-fluorouracil; GP, cisplatin plus gemcitabine

The P value was calculated using the Pearson χ^2^ test.
