# Supplementary figures and images for: Comparison of Gemcitabine Plus Cisplatin vs. Docetaxel Plus Fluorouracil Plus Cisplatin Palliative Chemotherapy for Metastatic Nasopharyngeal Carcinoma
Source: Front Oncol. 2020 Aug 6;10:1295. doi: 10.3389/fonc.2020.01295 (PMC7425654; doi:10.3389/fonc.2020.01295)

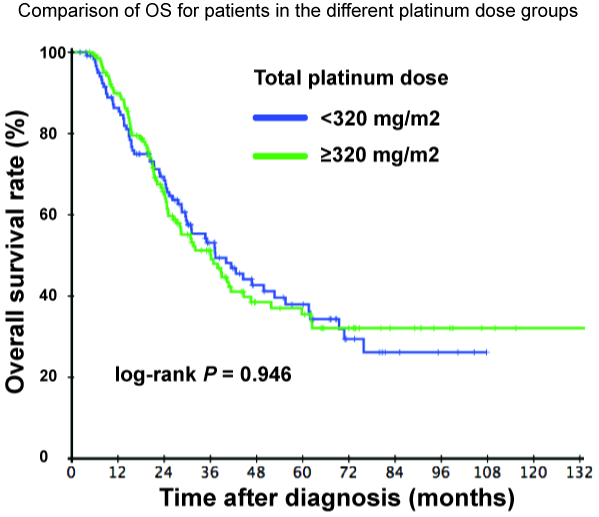

Supplement: Supplementary file 2 [file Image_1.tif]
